# Effect of Computer Screen Background Hues on Attention, Memory, and Visual Task Performance in Medical Students: A Repeated Measures Pilot Study

**DOI:** 10.7759/cureus.115960

**Published:** 2026-09-08

**Authors:** Mrinal Patnaik, Mohit Patnaik, Ravi Kaushik, Iesheta Patnaik, Neelam Vaney

**Affiliations:** 1 Forensic Medicine and Toxicology, All India Institute of Medical Sciences, Raipur, Raipur, IND; 2 Pharmacology, National Capital Region Institute of Medical Science, Meerut, IND; 3 Physiology, Maulana Azad Medical College, University of Delhi, New Delhi, IND; 4 Pediatric and Preventive Dentistry, Inderprastha Dental College and Hospital, Ghaziabad, IND; 5 Physiology, Amrita School of Medicine, Faridabad, IND

**Keywords:** cognition, computer screen, hues, medical students, task performance

## Abstract

Background: Digital screen characteristics may influence visual comfort, circadian physiology, and cognitive performance. Although blue-enriched light has been extensively studied, less is known about whether the background hue of a computer screen affects short-duration cognitive task performance. This study compared balanced white, red-predominant, and blue-predominant computer-screen conditions with printed white paper in medical students.

Methods: Thirty medical students completed four testing sessions one week apart in a repeated-measures design. The visual conditions were balanced white, red-predominant, and blue-predominant computer-screen hues, and printed white paper. Cognitive performance was assessed using an author-developed PQBD letter-identification task, a picture-difference task, comprehension-based multiple-choice questions (MCQs), and the Pittsburgh Sleep Quality Index (PSQI). Differences across conditions were analyzed using the Friedman test, with Wilcoxon signed-rank post-hoc comparisons and Holm correction. Kendall’s W was reported as an effect-size measure.

Results: Paper was associated with the highest performance on several tasks, while the red screen condition performed better than the balanced white screen on selected attention and recall outcomes. PQBD performance differed significantly across conditions (Friedman χ²(3) = 37.08, p < 0.001; Kendall’s W = 0.412). Mean correct-response percentages were 71.40 ± 17.31% for white screen, 81.26 ± 10.35% for red screen, 80.63 ± 9.51% for blue screen, and 89.92 ± 11.54% for paper. Picture-difference performance also differed significantly (χ²(3) = 26.15, p < 0.001; W = 0.291), as did comprehension-based MCQ performance (χ²(3) = 52.03, p < 0.001; W = 0.578).

Conclusion: Short-duration cognitive task performance differed across visual conditions in this exploratory repeated-measures study. Printed paper showed the highest performance on several tasks, while red-predominant screen hue was associated with better performance than balanced white on selected outcomes. Because the study used a small sample, fixed condition order, exploratory cognitive measures, and software-based rather than spectroradiometrically calibrated display settings, the findings should be interpreted cautiously. It appears that background hues of computer screens can affect task performance involving memory and concentration capabilities of medical students. Paper should be preferred in tasks requiring cognitive function, including memory and attention.

## Introduction

In the past few decades, there has been a more significant change in how we access information than in the preceding 600 years since the printing press's invention in the 1440s [1]. The "penetration," i.e., the duration for the widespread adoption and use of new products or inventions, has been decreasing rapidly in electronic media [2]. Over the past two decades, digital devices have increasingly changed the way educational material is accessed and consumed. Healthcare professionals and medical students now routinely use computers, tablets, and smartphones for reading, communication, and academic work. As digital learning becomes more common, characteristics of electronic displays, including brightness and color composition, may influence visual comfort, sleep-related physiology, and cognitive performance.

Prolonged exposure to light emitted from electronic displays has been associated with changes in circadian physiology, particularly when exposure occurs during the evening or night. There has been considerable research concluding that long exposure to glare from electronic screens causes definite changes in melatonin secretion and circadian rhythm, affecting sleep cycles. It has been shown that screens affect higher brain functions; thus, performance of ordinary tasks and academic performance may be affected [3-8].

As people spend more time in front of bright screens, particularly before bedtime, issues such as delayed sleep, reduced sleep duration, and impaired sleep quality increase. Short-wavelength, blue-enriched light has been shown to suppress melatonin secretion and alter circadian timing [7,9]. To combat this, technological adaptations have been made, such as reducing screen brightness and shifting the screen hue to a lower temperature, to make nighttime use of electronic screens less harmful. Various applications, such as Twilight^TM^ and flux^TM^, have been developed based on these findings that reduce blue-weighted light and shift screen color temperature toward warmer hues during evening use [10,11].

In 2014, Fossum et al. reported that mobile phone use before sleep was associated with insomnia symptoms, daytime sleepiness, and reduced morning alertness [3]. Exposure to artificial light in the evening can also disrupt normal melatonin signaling and alter circadian physiology [4]. The spectral characteristics and color temperature of light may further influence alertness and cognitive performance. Chellappa et al. demonstrated that exposure to light of different spectral composition can affect melatonin secretion, subjective alertness, and selected measures of cognitive performance [12]. Similarly, the Sleep Time-Related Information and Communication Technology (STRICT) study reported associations between nighttime screen use, insomnia, later bedtimes, shorter sleep duration, and poorer daytime functioning [7].

Apart from its effects on sleep and circadian physiology, the color characteristics of visual displays may influence cognitive task performance. Hatta et al. evaluated performance under red, blue, and beige computer-display conditions and found that the effect of display color varied according to the type and difficulty of the task [13]. Their findings suggest that the relationship between display hue and cognitive performance may be task-dependent rather than uniform. Although alterations in computer-display color are not equivalent to controlled exposure to monochromatic or ambient light sources, existing evidence supports further investigation into whether screen background hues influence attention, visual discrimination, and short-duration cognitive performance.

Sleep itself plays an important role in learning and memory. Extensive experimental evidence has demonstrated that sleep contributes to memory consolidation and that sleep and wakefulness are associated with different stages and mechanisms of memory processing [14]. Consequently, sleep quality represents an important potential factor when studying attention, recall, and other cognitive outcomes in students who frequently use electronic devices.

Previous experimental work has also shown that different wavelengths of light can produce biological effects at the cellular level, including responses to red light-emitting diode exposure [15]. However, such cellular effects cannot be directly extrapolated to cognitive responses during computer use. More relevantly, studies of artificial lighting have demonstrated that spectral composition and color temperature can influence alertness, visual comfort, and cognitive performance [12]. Despite these observations, comparatively limited evidence is available regarding the effects of computer-screen background hues on short-duration attention, visual discrimination, and memory-related task performance.

Therefore, the present exploratory repeated-measures study was conducted to compare cognitive task performance under balanced white, red-predominant, and blue-predominant computer-screen conditions and printed white paper in medical students. The study specifically assessed visual attention and discrimination, short-term recall, and self-reported sleep quality. We hypothesized that performance would differ across visual conditions and that the effect of screen hue might vary according to the cognitive demands of the task.

## Materials and methods

Ethical clearance

Ethical approval was obtained from the Institutional Ethics Committee for Human Research (IEC-HR) prior to participant recruitment for the student project vide ICMR-STS reference number: 2016-07047. To ensure confidentiality, personal and demographic data were recorded on paper forms and securely stored by the principal investigator.

Study design

A prospective observational, exploratory, repeated measures study among medical student volunteers. Eligibility criteria included English literacy, 6/6 visual acuity with or without glasses, absence of blue-light filtering prescription glasses, absence of alcohol use, nonsmokers, no drug use, no caffeine abuse, no physical infirmity, and no diagnosed chronic illnesses. Each participant underwent testing under four visual conditions on four separate occasions, with sessions conducted one week apart. Measures included performance in a set of cognition tests as described below.

Recruitment of participants

Participants were recruited through rolling sampling by word of mouth. Written informed consent was obtained from all participants, and a copy was provided to each volunteer. Participants were informed that participation was voluntary and that they could withdraw from the study at any time without consequence

Sample size

The study was designed as an exploratory pilot study. No a priori power calculation was performed. Thirty-three participants were recruited, of whom 30 completed all four sessions. The sample should therefore be interpreted as a pragmatic exploratory sample rather than a formally powered cohort.

Testing procedures

Each participant underwent testing under four visual conditions on four separate occasions, with sessions conducted one week apart. Each session lasted approximately 15-20 minutes. The four conditions were a white computer screen with balanced red-green-blue (RGB) settings, a red-predominant computer screen hue, a blue-predominant computer screen hue, and a printed white paper. The principal investigator, a peer of the participants, conducted and supervised all sessions. The experimental conditions were administered in a fixed sequence (Figure 1). The sequence was not randomized or counterbalanced. 

**Figure 1:**
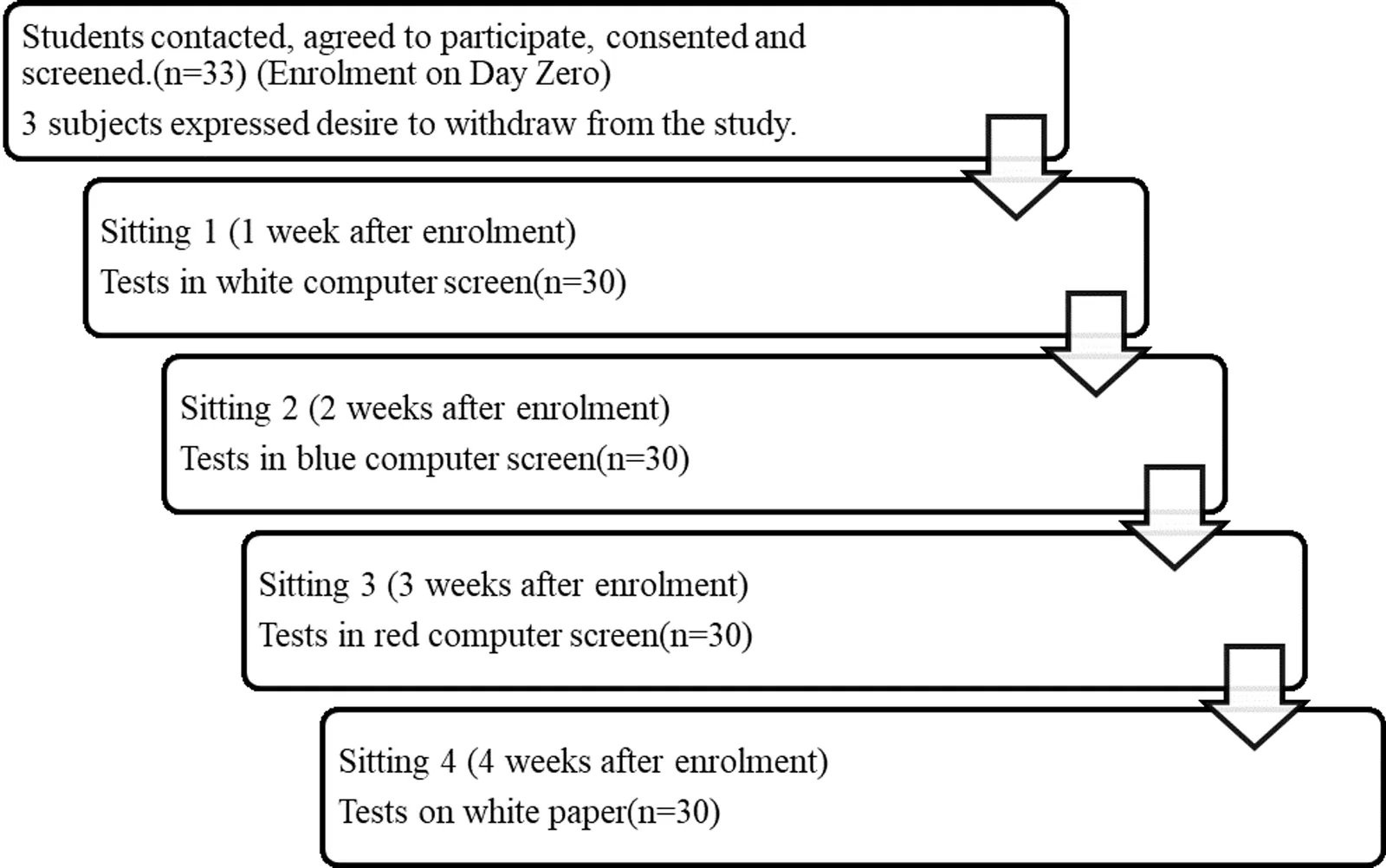
Flowchart showing the sequence of the study methodology This figure outlines participant recruitment, screening, and sequential testing sessions conducted one week apart under four conditions-white, red, and blue computer screen hues, and printed white paper

Following enrollment, a WhatsApp group was created to facilitate communication and scheduling of the weekly study sessions. Testing was conducted in the Medical Education Unit (MEU). Each participant completed a battery of tasks designed to assess attention, visual discrimination, short-term memory, recall, and sleep quality. The sequence of tasks during the computer-based sessions was standardized (Figure 2) as follows: PQBD grid test, pictorial difference game, comprehension-based MCQs, and PSQI questionnaire. 

**Figure 2:**
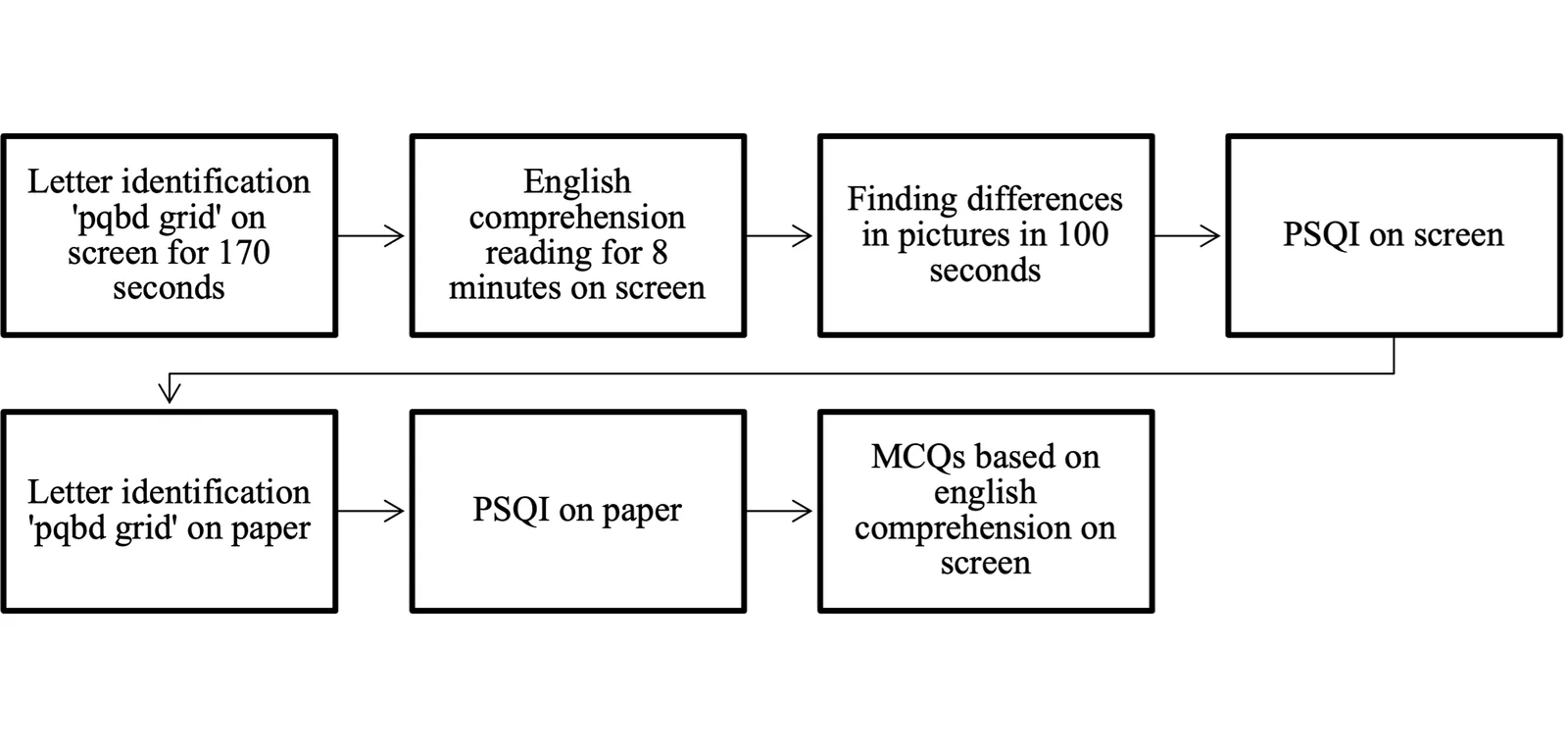
Sequence of tests in the test battery PSQI: Pittsburgh Sleep Quality Index; MCQs: multiple-choice questions The schematic depicts the order of tasks administered during each session: The letter identification "PQBD" grid test → English comprehension passage reading → picture difference task → PSQI questionnaire → recall-based MCQs. The arrangement ensured consistent cognitive load and time distribution across color settings

The tests included a letter identification test, reading an English comprehension exercise, finding differences in pictures through a “spot-the-differences” task on an online free gaming website [16-18] to assess visual attention and discrimination, and an on-screen questionnaire (Pittsburgh Sleep Quality Index, PSQI) [19]. This sequence was maintained across the computer-screen conditions to provide a consistent cognitive workload and approximate time distribution across sessions. 

Letter-identification task

Attention and visual discrimination were assessed using an author-developed letter-identification task consisting of the letters “p,” “q,” “b,” and “d.” The computerized version was developed by the authors as a Java application (Figure 3).

**Figure 3:**
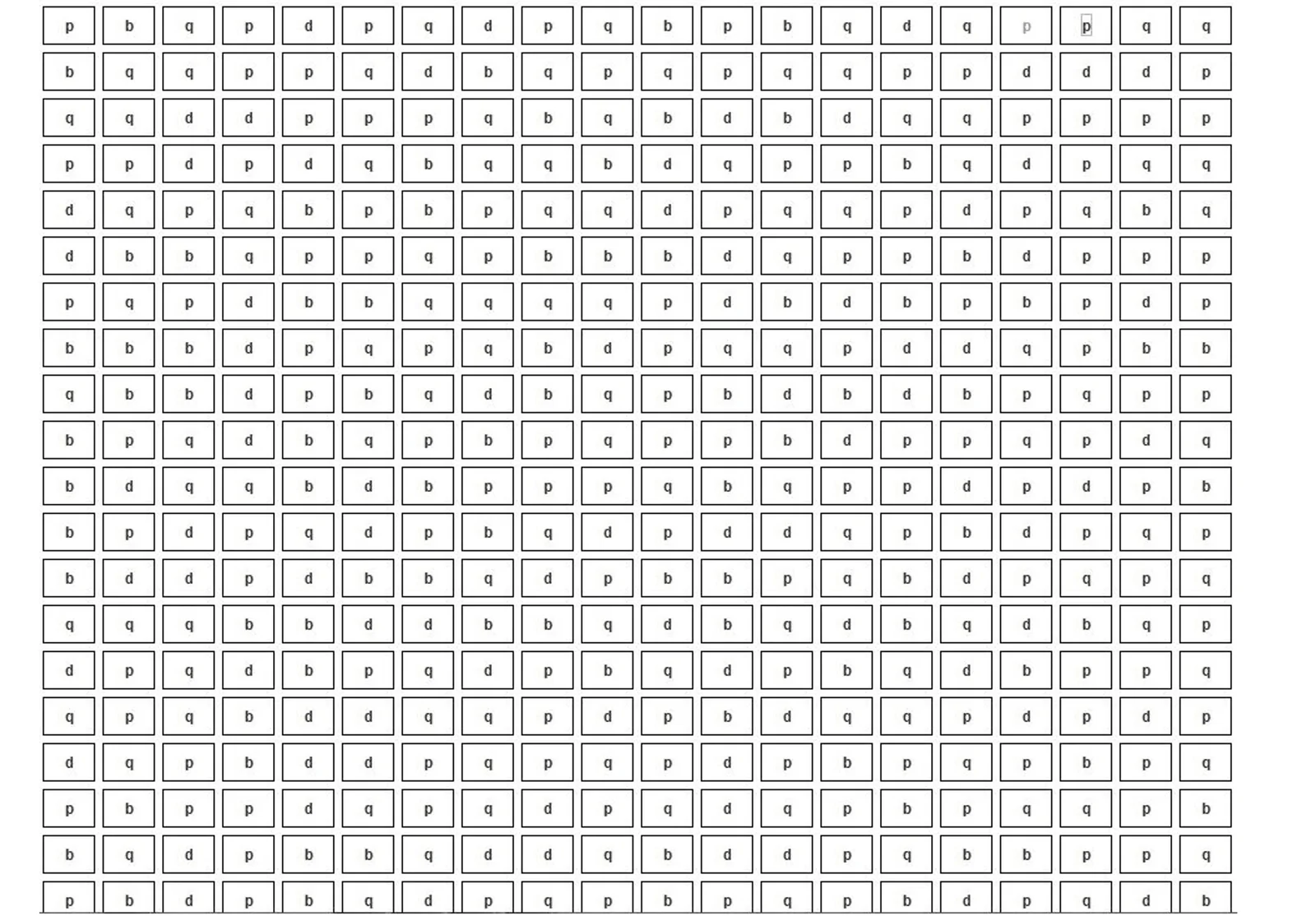
Image showing the letter identification test (PQBD grid) in the java application Letter identification test: developed by the authors

The application displayed 400 buttons arranged in a 20 × 20 grid, with the letters “p,” “q,” “b,” and “d” presented in a randomized sequence. The letters were displayed using Arial font, size 14. Participants were required to identify the designated target letters within the allotted time. The application automatically terminated after 170 seconds. The number of responses, number of errors, number of items remaining, and percentage of correct responses were recorded. A corresponding paper version of the task was also administered in the "paper sitting."

The PQBD task was developed specifically for this study and was not a previously standardized psychometric instrument. Accordingly, its findings are interpreted as exploratory measures of visual attention and discrimination rather than as results from a formally validated neuropsychological test.

English comprehension and recall task

Participants were asked to read an English comprehension passage for approximately eight minutes. The passage was selected to have a level of comprehension and difficulty comparable to the Central Board of Secondary Education (CBSE) Class XII English Core curriculum examination materials available for public educational use [20].

Short-term retention and recall were subsequently assessed using five multiple-choice questions (MCQs) based on the information contained in the comprehension passage. Each question contained four response options. The total number of correct responses, ranging from 0 to 5, was recorded for analysis.

Picture-difference task

Visual attention, scanning, and discrimination were assessed using a “spot-the-difference” picture task obtained from freely available online game resources [16-18]. Participants were required to identify differences between paired images within the predetermined task period. The number of correctly identified differences and the number of differences not identified were recorded. This activity was used as an exploratory visual-attention task and was not treated as a standardized neuropsychological instrument.

Assessment of sleep quality

Sleep quality was assessed using the PSQI, a validated self-report instrument designed to evaluate sleep quality over the preceding month [19]. The PSQI global score was calculated according to the published scoring instructions. PSQI assessment was included to characterize participants' recent sleep quality and to permit exploratory assessment of the relationship between sleep quality and cognitive-task performance.

Computer-screen display conditions

The study used software-based RGB, brightness, contrast, and gamma adjustments. Absolute luminance, wavelength-specific output, and spectral power distribution were not independently measured. The computer display was adjusted through operating-system settings to generate balanced white, red-predominant, and blue-predominant screen conditions, as detailed in Table 1.

**Table 1:** Color settings used for visual display conditions RGB: red, green, blue The table lists the values applied to configure the computer screen hues for white, red, and blue visual conditions

	RGB components	Red	Blue	Green
White/balanced hue	Gamma	1.0	1.0	1.0
	Brightness	0	0	0
	Contrast	50	50	50
Red-predominant hue	Gamma	1.0	1.0	1.0
	Brightness	40	-60	-60
	Contrast	50	40	40
Blue-predominant hue	Gamma	1.0	1.0	1.0
	Brightness	-60	40	-60
	Contrast	40	50	40

Control of potential confounding variables

To reduce variation due to circadian effects, the four weekly testing sessions for each participant were conducted at approximately the same time of day. However, the study did not formally quantify or standardize other potential short-term confounding variables, including caffeine intake immediately before individual testing sessions, duration of recent screen exposure, ambient light intensity, and recent physical activity. Because every participant completed testing under all four conditions, each participant served as his or her own comparison across experimental conditions. These factors are, however, still acknowledged as potential residual confounders.

Data collection and statistical analysis

The principal outcome measures were percentage of correct responses in the PQBD letter-identification task, number of pictorial differences correctly identified, number of correct responses in the five-question comprehension-based MCQ assessment, and PSQI global score. Errors and uncompleted responses in the letter identification test and picture-difference tasks were also recorded as supplementary descriptive measures.

Because the same 30 participants underwent testing under all four experimental conditions, the observations represented repeated measures rather than independent groups. Given the relatively small sample size and the discrete or bounded nature of several outcome measures, repeated-measures nonparametric analyses were used. Continuous variables are summarized as mean ± standard deviation (SD). Differences across the four experimental conditions were evaluated using the Friedman test. When the overall Friedman test demonstrated a statistically significant difference, post-hoc pairwise comparisons were performed using the Wilcoxon signed-rank test. Holm adjustment was applied to pairwise p-values to account for multiple comparisons.

Effect size for the overall Friedman test was expressed using Kendall’s coefficient of concordance (Kendall’s W). A two-sided p-value < 0.05 was considered statistically significant. Statistical analysis was performed using a licensed copy of MS Excel (Microsoft Corporation, Redmond, Washington, United States) and IBM SPSS Statistics for Windows, Version 23 (Released 2015; IBM Corp., Armonk, New York, United States).

## Results

Thirty participants completed all four study sessions and were included in the final analysis. Cognitive performance was compared across four conditions: a balanced white computer screen, a red-predominant computer screen, a blue-predominant computer screen, and a printed white paper.

Letter-identification task

Table 2 displays the percentage of correct responses in the PQBD letter identification task. Performance in the PQBD letter-identification task differed significantly across the four visual conditions. The mean percentage of correct responses was 71.40 ± 17.31% under the white screen condition, 81.26 ± 10.35% under the red screen condition, 80.63 ± 9.51% under the blue screen condition, and 89.92 ± 11.54% during the paper condition.

**Table 2:** Performance in different sittings in letter identification test SD: standard deviation; CI: confidence interval

	Paper	Red	Blue	White
Correct responses: percentage (mean ± SD)	89.92 ± 11.54 (95% CI: 85.612-94.227)	81.26 ± 10.35 (95% CI: 77.393-85.123)	80.63 ± 9.51 (95% CI: 77.076-84.179)	71.40 ± 17.31 (95% CI: 64.934-77.862)
Mistakes: numbers (mean ± SD)	0.033 ± 0.183 (95% CI: 0-0.098)	0.767 ± 1.402 (95% CI: 0.244-1.290)	0.467 ± 0.730 (95% CI: 0.217-0.717)	0.633 ± 0.890 (95% CI: 0.374-0.892)

The Friedman test demonstrated a statistically significant overall difference across the four conditions (χ²(3) = 37.08; p < 0.001), with a Kendall’s W of 0.412, indicating a moderate-to-large condition effect. Post-hoc Wilcoxon signed-rank tests with Holm adjustment showed that performance on paper was significantly higher than that under the white screen, red screen, and blue screen conditions (adjusted p < 0.001, p = 0.001, and p = 0.001, respectively). Both the red and blue screen conditions were also associated with significantly higher correct-response percentages than the white screen condition (adjusted p = 0.020 for both comparisons). No statistically significant difference was observed between the red and blue screen conditions (adjusted p = 0.673). Thus, for the PQBD task, the highest mean performance was observed on paper, followed by the red and blue screen conditions, with the lowest performance under the balanced white screen condition.

Picture-difference task

The mean number of pictorial differences correctly identified was 5.53 ± 1.46 under the white screen condition, 5.17 ± 1.42 under the red screen condition, 3.97 ± 1.50 under the blue screen condition, and 6.30 ± 1.52 under the paper condition. There was a statistically significant overall difference in performance across the four visual conditions (Friedman χ²(3) = 26.15; p < 0.001; Kendall’s W = 0.291).

Post-hoc comparisons demonstrated that the blue screen condition was associated with significantly lower performance than the white screen (adjusted p = 0.001), red screen (adjusted p = 0.015), and paper conditions (adjusted p < 0.001). Performance on paper was also significantly higher than that under the red screen condition (adjusted p = 0.028). The differences between white and red screen conditions and between white screen and paper were not statistically significant after Holm correction. In contrast to the PQBD task, the picture-difference task showed the highest mean performance on paper, followed by the white and red screen conditions, with the lowest performance under the blue screen condition.

Comprehension-based MCQs

The mean number of correct answers out of five was 2.10 ± 1.12 under the white screen condition, 3.70 ± 0.92 under the red screen condition, 2.23 ± 1.33 under the blue screen condition, and 4.33 ± 1.06 during the paper condition. The Friedman test demonstrated a statistically significant overall difference in MCQ performance across the four conditions (χ²(3) = 52.03; p < 0.001), with a Kendall’s W of 0.578, indicating a relatively large condition effect.

Post-hoc analysis showed that the paper condition was associated with significantly higher MCQ scores than the white screen, blue screen, and red screen conditions (adjusted p < 0.001; p < 0.001; and p = 0.005, respectively). The red screen condition was also associated with significantly higher scores than both the white and blue screen conditions (adjusted p < 0.001 for both comparisons). There was no statistically significant difference between the white and blue screen conditions (adjusted p = 0.607). Overall, performance on the comprehension-based MCQs followed the paper screen, then red screen, with the white and blue screen conditions showing lower performance.

Pittsburgh Sleep Quality Index (PSQI)

The mean PSQI global scores were 6.27 ± 2.90 during the white screen session, 5.53 ± 2.79 during the red screen session, 5.53 ± 2.80 during the blue screen session, and 5.90 ± 2.76 during the paper session. There was a small but statistically significant overall variation in PSQI scores across the four sessions (Friedman χ²(3) = 15.12; p = 0.002; Kendall’s W = 0.168). However, none of the individual pairwise comparisons remained statistically significant after Holm correction for multiple testing. Therefore, although PSQI scores showed modest overall variation across study sessions, no specific pair of sessions demonstrated a statistically significant difference after correction for multiple comparisons.

Summary of cognitive outcomes

The repeated-measures analyses demonstrated statistically significant differences across visual conditions for the PQBD letter-identification task, picture-difference task, and comprehension-based MCQs. Printed white paper was associated with the highest mean performance on all three cognitive outcomes. The red-predominant screen condition performed better than the balanced white screen condition for the PQBD and MCQ tasks, whereas the blue-predominant screen condition showed the lowest mean performance on the picture-difference task.

As shown in Table 3, statistically significant overall differences were observed across the four visual conditions for the PQBD task, picture-difference task, comprehension-based MCQs, and PSQI scores. The largest condition effect was observed for the MCQ outcome (Kendall’s W = 0.578), followed by the PQBD task (W = 0.412), picture-difference task (W = 0.291), and PSQI scores (W = 0.168).

**Table 3:** Overall repeated-measures comparison of cognitive performance and PSQI scores across the four visual conditions PQBD: letter-identification task using the letters p, q, b, and d; MCQ: multiple-choice question; PSQI: Pittsburgh Sleep Quality Index Values are presented as mean ± standard deviation. Overall differences across the four repeated-measures conditions were assessed using the Friedman test. Kendall’s W was used as the effect-size measure for the Friedman test. A two-sided p-value < 0.05 was considered statistically significant

Outcome	White screen	Red screen	Blue screen	Paper	Friedman χ²	p-value	Kendall’s W
PQBD correct responses (%)	71.40 ± 17.31	81.26 ± 10.35	80.63 ± 9.51	89.92 ± 11.54	37.08	<0.001	0.412
Picture differences identified	5.53 ± 1.46	5.17 ± 1.42	3.97 ± 1.50	6.30 ± 1.52	26.15	<0.001	0.291
MCQs correct (out of 5)	2.10 ± 1.12	3.70 ± 0.92	2.23 ± 1.33	4.33 ± 1.06	52.03	<0.001	0.578
PSQI global score	6.27 ± 2.90	5.53 ± 2.79	5.53 ± 2.80	5.90 ± 2.76	15.12	0.002	0.168

Table 4 presents the post-hoc pairwise comparisons. For the PQBD task, paper significantly outperformed all three screen conditions, while both red and blue screen conditions performed significantly better than the balanced white screen condition. For the picture-difference task, the blue screen condition showed significantly poorer performance than the white, red, and paper conditions. For comprehension-based MCQs, paper significantly outperformed all screen conditions, while the red screen condition also performed significantly better than the white and blue screen conditions.

**Table 4:** Post-hoc pairwise comparisons between visual conditions for cognitive performance outcomes PQBD: letter-identification task using the letters p, q, b, and d; MCQ: multiple-choice question Post-hoc pairwise comparisons were performed using the Wilcoxon signed-rank test after a statistically significant Friedman test. Holm adjustment was applied to account for multiple comparisons. Adjusted p-values are reported. A two-sided adjusted p-value < 0.05 was considered statistically significant

Outcome	Comparison	Holm-adjusted p-value
PQBD	White vs red	0.02
PQBD	White vs blue	0.02
PQBD	White vs paper	<0.001
PQBD	Red vs blue	0.673
PQBD	Red vs paper	0.001
PQBD	Blue vs paper	0.001
Picture-difference task	White vs red	0.334
Picture-difference task	White vs blue	0.001
Picture-difference task	White vs paper	0.229
Picture-difference task	Red vs blue	0.015
Picture-difference task	Red vs paper	0.028
Picture-difference task	Blue vs paper	<0.001
MCQs	White vs red	<0.001
MCQs	White vs blue	0.607
MCQs	White vs paper	<0.001
MCQs	Red vs blue	<0.001
MCQs	Red vs paper	0.005
MCQs	Blue vs paper	<0.001

## Discussion

The present exploratory repeated-measures study showed that short-duration cognitive performance varied across visual conditions, and the pattern differed by task. Printed white paper produced the highest mean performance across the three cognitive outcomes, while the red-predominant screen condition showed relatively favorable performance in the PQBD letter-identification and comprehension-based recall tasks. These findings suggest that the effect of screen hue may depend on the perceptual and cognitive demands of the activity rather than on hue alone.

Letter-identification task

PQBD performance differed significantly across the four conditions, with the highest mean percentage of correct responses on paper, followed by the red- and blue-predominant screens, and the lowest performance under the balanced white screen. The overall effect size was moderate to large.

The PQBD task required rapid discrimination among visually similar letters within a restricted visual field. Differences in contrast, glare, luminance, visual familiarity, and comfort may therefore have contributed to performance. Paper also differs fundamentally from an illuminated display because it relies on reflected rather than emitted light.

Both red and blue screen conditions performed significantly better than the balanced white screen, while red and blue did not differ significantly from each other. Hatta et al. similarly reported that the effect of computer-display color varied according to the type and difficulty of the task [13]. Berson et al. demonstrated that a subset of retinal ganglion cells is intrinsically photosensitive and contributes to non-image-forming responses to light, particularly circadian photoreception [21]. This finding established a physiological pathway through which light characteristics can influence biological functions beyond conventional visual perception. More recent work by Yang et al. showed that blue-light backgrounds could slow exogenous visual-attention shifts without producing the same effect on endogenous attention [22]. Vani et al. also reported differences in attention-related performance under different colored-light conditions, with relatively favorable findings under white and red illumination [23]. Although these studies differ from the present experiment in their light source, spectral characteristics, and task design, they support the interpretation that color-related effects on attention are task-dependent.

Picture-difference task

The picture-difference task showed a different pattern. Paper produced the highest mean score, followed by the white and red screen conditions, while the blue-predominant screen produced the lowest performance. The blue condition was significantly worse than the white, red, and paper conditions after correction for multiple comparisons.

This task required repeated scanning of a larger visual field and comparison of multiple details, which may explain why its response to hue differed from the more spatially restricted PQBD task. The findings of Yang et al., in which blue-light backgrounds impaired exogenous visual-attention shifts [22], provide a possible context for this observation because the picture task also required repeated redirection of attention.

However, blue light does not consistently impair cognition. Lee et al. found no significant behavioral effect of blue-light exposure across sustained-attention, task-switching, and Stroop paradigms [24]. Taken together with Hatta et al. [13], these studies suggest that display-color effects are likely to depend on task demands rather than being uniformly beneficial or harmful. The picture-difference task used in the present study was exploratory and not a standardized neuropsychological instrument [16-18]; therefore, these findings should be considered preliminary.

Comprehension-based recall

Comprehension-based MCQ performance also differed significantly across conditions. Paper showed the highest mean score, followed by the red-predominant screen, whereas the white and blue conditions produced lower scores. The overall condition effect was relatively large.

The red screen condition significantly outperformed both the balanced white and blue conditions. Chellappa et al. previously reported that blue-enriched light can influence alertness and selected cognitive outcomes [12]. In contrast, our recall task showed better performance under the red-predominant condition. This difference may reflect variations in exposure duration, spectral composition, task structure, and the distinction between ambient-light exposure and alteration of a computer-screen background. The absence of a consistent blue-light benefit in the study by Lee et al. [24], together with the task-dependent findings of Hatta et al. [13], further supports the view that no single display hue is likely to optimize all forms of cognitive performance.

Sleep quality

PSQI scores showed a small but statistically significant overall variation across sessions, although no individual pairwise comparison remained significant after correction for multiple testing. Sleep has an established role in memory formation and consolidation [14]. However, comparison of PSQI scores across sessions does not directly establish whether sleep quality affected cognitive performance. Furthermore, sleep was assessed using a self-reported questionnaire rather than objective measures such as actigraphy or polysomnography.

Accordingly, the present findings should not be interpreted as showing that sleep quality has no effect on cognition or that participants compensated for poor sleep. Future studies should examine the direct relationship between objective sleep measures and cognitive performance under different display conditions.

Physiological and perceptual considerations

Intrinsically photosensitive retinal ganglion cells contribute to circadian and non-image-forming responses to light [21], and short-wavelength light has been extensively studied in relation to melatonin suppression, alertness, and cognition [12,21]. However, recent research indicates that behavioral responses to colored light are more complex than a simple wavelength-response relationship.

Yang et al. found that the effect of a blue-light background on exogenous attention could not be explained solely by selective stimulation of blue-sensitive photoreceptor pathways [22], while Lee et al. found no consistent behavioral benefit of blue light [24]. Our intervention consisted of software-based alteration of screen hue rather than calibrated monochromatic-light exposure. Therefore, the present study cannot establish melatonin suppression, ipRGC activation, or another specific physiological mechanism as the cause of the observed differences. Such mechanisms could be regarded as possible explanatory frameworks.

Visual fatigue may also contribute to performance during screen-based tasks, although it was not directly measured in this study. Xie et al. reported that screen color mode and color temperature influenced subjective and objective indices of visual fatigue, with effects varying according to ambient illumination [25].

This is relevant because differences in visual comfort could potentially affect task performance. Visual fatigue remains a possible explanation, and further studies could include ambient illuminance, blink rate, pupil diameter, and subjective eye strain, which were not assessed in the present study.

Overall interpretation

Across the three cognitive tasks, paper produced the highest mean performance, but this should not be interpreted as evidence that paper is universally superior to electronic displays. Paper and screens differ in multiple characteristics beyond hue, including reflected versus emitted light, luminance, glare, contrast, and physical presentation.

Among the screen conditions, the red-predominant hue showed relatively favorable performance in the PQBD and recall tasks, whereas the blue-predominant hue performed particularly poorly in the picture-difference task. The differing patterns across tasks support a task-dependent effect of display color, consistent with both Hatta et al. [13] and more recent studies [22-24]. Overall, the findings suggest that the relationship between display color and cognitive performance depends on the interaction between visual characteristics of the display and the cognitive demands of the task, rather than on a universally optimal screen hue.

Limitations

This study has several limitations. It was an exploratory pilot study with a small, homogeneous sample of medical students, limiting generalizability. The experimental sequence was not randomized or counterbalanced, so learning, practice, habituation, fatigue, and order effects may have influenced performance.

The PQBD and picture-difference tasks were exploratory and not standardized neuropsychological instruments. Although sessions were conducted at approximately the same time of day and several potential confounders were addressed through eligibility criteria, recent caffeine intake, prior screen exposure, physical activity, and ambient illumination were not formally quantified.

Sleep quality was assessed using the self-reported PSQI rather than objective measures such as actigraphy [19]. In addition, screen conditions were created using software-based RGB, brightness, contrast, and gamma adjustments, while absolute luminance, spectral power distribution, and retinal irradiance were not measured. Larger randomized and counterbalanced studies using calibrated displays, validated cognitive measures, objective visual-fatigue assessments, and objective sleep measurements are needed to determine whether these findings are reproducible.

## Conclusions

In this exploratory repeated-measures study, cognitive task performance differed across visual conditions. Printed white paper was associated with the highest mean performance on the letter-identification, picture-difference, and comprehension-based recall tasks. Among the screen conditions, the red-predominant hue showed relatively favorable performance in the letter-identification and recall tasks, whereas the blue-predominant hue showed the lowest performance in the picture-difference task. These findings suggest that the effect of screen hue may vary according to the visual and cognitive demands of the task rather than reflecting a universally optimal display color. However, the small homogeneous sample, fixed testing sequence, use of exploratory cognitive measures, and absence of direct spectral calibration limit the strength and generalizability of the conclusions.
